# Effect of Obesity and Osteocalcin on Brain Glucose Metabolism in Healthy Participants

**DOI:** 10.3390/brainsci13060889

**Published:** 2023-05-31

**Authors:** Seunghyeon Shin, Hyun-Yeol Nam

**Affiliations:** Department of Nuclear Medicine, Samsung Changwon Hospital, Sungkyunkwan University School of Medicine, Changwon 06351, Republic of Korea; forladou@naver.com

**Keywords:** fluorine-18-fluorodeoxyglucose, positron emission tomography–computed tomography, obesity, osteocalcin, brain

## Abstract

We evaluated the effects of obesity and osteocalcin on glucose metabolism in the brain. A total of 179 healthy men were enrolled in this study. After preprocessing positron emission tomography images, including by performing coregistration, spatial normalization, and smoothing, regression analysis was conducted to identify the correlation between body mass index, osteocalcin, and brain glucose metabolism. Body mass index was positively correlated with brain glucose metabolism in the anterior lobe of the right cerebellum, the anterior and posterior lobes of the left cerebellum, the right middle frontal gyrus (Brodmann area 9), the right cingulate gyrus (Brodmann area 32), the right anterior cingulate (Brodmann area 32), the left middle frontal gyrus (Brodmann area 10), and the subgyral area of the left frontal lobe. Osteocalcin was negatively correlated with glucose metabolism in the anterior lobe of the left cerebellum. Body mass index was positively correlated with brain glucose metabolism in the prefrontal cortex and cerebellum. Osteocalcin levels were negatively correlated with brain glucose metabolism in the left cerebellum.

## 1. Introduction

The prevalence of obesity, which is defined as excessive fat accumulation, has increased worldwide [1]. Obesity is categorized using an individual’s body mass index (BMI, kg/m^2^), which is typically classified into the following three categories [2]: normal weight (18.5–24.9 kg/m^2^); overweight (equal to or higher than 25.0 kg/m^2^); and obese (equal to or greater than 30.0 kg/m^2^) [2]. Obesity is also known to be associated with type 2 diabetes mellitus (DM), fatty liver disease, hypertension, myocardial infarction, stroke, osteoarthritis, Alzheimer’s disease, depression, and malignancy [1].

Cognitive function is defined as the processing, integration, storage, and retrieval of information, and perception, attention, memory, and executive functions are types of cognitive functions [3]. Numerous studies have reported that obesity is associated with impaired cognitive function. Among obese children and adolescents, cognitive deficits are observed in executive function, short-term memory, global functioning, and verbal abilities [3]. Among obese adults, poor performance in global cognitive function, memory, language, and motor skills is associated with higher BMI [3]. This cognitive dysfunction is independent of socioeconomic, depressive, and cardiovascular factors [3]. Obesity is also known to increase the risk of mild cognitive impairment, dementia, and Alzheimer’s disease [4]. One study reported that the relative risks of the development of dementia and Alzheimer’s disease among middle-aged obese and overweight participants were 2.04 and 1.64, respectively [4].

2-deoxy-2-(fluorine-18)fluoro-D-glucose (F-18 FDG) positron emission tomography (PET)/computed tomography (CT) is widely used in oncology, neurology, and psychiatry [5,6]. F-18 FDG PET is useful in differentiating between various types of primary dementia and predicting conversion from MCI to Alzheimer’s disease [7]. As obesity is known to be associated with cognitive dysfunction and the risk of mild cognitive impairment and Alzheimer’s disease, F-18 FDG PET/CT might be capable of identifying the difference between obese and normal-weight participants. Thus, various PET studies have been conducted to identify the brain changes associated with obesity. However, these studies have reported inconsistent results [8,9,10]. Due to these inconsistent results, we aimed to identify the effect of obesity on brain glucose metabolism within a large cohort of middle-aged participants. Several factors, such as aging, obesity, DM, and insulin resistance, are known to affect glucose metabolism in the brain [11]. Although osteocalcin is known to play a role in brain development and cognitive function [12], no studies have evaluated the correlation between human brain glucose metabolism and osteocalcin. Therefore, we aimed to evaluate the effect of osteocalcin on glucose metabolism in the human brain.

## 2. Subjects and Methods

### 2.1. Participants

Data concerning participants who had undergone general health examinations, including F-18 FDG PET/CT scans and bone mineral density (BMD) tests, at our institute between January 2013 and December 2013 were retrospectively reviewed. Participants who had a clinically diagnosed neurodegenerative disease, psychiatric disease, or previous cerebrovascular accident were excluded. Participants who used any neuropsychological medication were also excluded. As the majority of the enrolled participants were men, we included only men in the study. Anthropometric measurements of the participants were taken. Using the measured heights and weights, BMI was calculated by dividing the weight (kg) by the square of the height (m) for each participant. After overnight fasting, blood samples were collected to measure fasting blood glucose, glycated hemoglobin (HbA1C), and osteocalcin levels, which were determined using standard laboratory methods. BMD of the lumbar spine and femur was measured using dual-energy X-ray absorptiometry. According to the World Health Organization’s classification, we defined normal density, osteopenia, and osteoporosis as follows: normal (T-score ≥ −1.0), osteopenia (−2.5 < T-score < −1.0), and osteoporosis (T-score ≤ −2.5). This study was approved by the institutional review board of our institute, and the requirement for informed consent was waived due to the retrospective design of the study.

### 2.2. Imaging Protocol

Participants were intravenously injected with F-18 FDG at a dose of 3.7 MBq/kg (0.1 mCi/kg) body weight. PET/CT was performed 60 min after injection using an integrated PET/CT scanner (Discovery 710, GE Healthcare, Waukesha, WI, USA). During image acquisition, a CT scan covering the area from the vertex of the skull to the proximal thigh was initially carried out for attenuation correction with a slice thickness of 3.75 mm (120 kV). PET data were obtained using a high-resolution whole-body scanner with an axial field of 15.7 cm. The PET images were reconstructed using an iterative algorithm (VUE-Point FX, iteration: 2, subsets: 16), with an image matrix size of 128 × 128.

### 2.3. Image Analysis

Using Amide’s Medical Image Data Examiner program, the entire skull area was extracted from each of the original PET and CT scans and converted into a NIFTI file format. Statistical Parametric Mapping 12 (Wellcome Department of Imaging Neuroscience, Institute of Neurology, University College London) was implemented in MATLAB R2020b (MathWorks, Natick, MA, USA) for preprocessing. First, coregistration of PET images and corresponding CT images was performed for each participant. Second, spatial normalization of PET images into a standard Montreal Neurological Institute (MNI) template was conducted using the deformation field of the CT image. As magnetic resonance imaging (MRI) of the brain was not performed for the enrolled participants, nonenhanced CT images were used for normalization because a previous study reported a high level of concordance between MRI and CT-based normalization [13]. Third, smoothing of the normalized PET image was performed using an FWHM filter with a voxel size of 8 mm × 8 mm × 8 mm. A regression analysis was conducted to identify the correlation between BMI and brain glucose metabolism. Age was also included as a covariate. Results were displayed when the family-wise error (FWE) corrected *p*-value was less than 0.05 and the minimum cluster size was 100 contiguous voxels. Regression analysis was also conducted using osteocalcin. When osteocalcin levels were within the normal range, we hypothesized that even if there was an effect on brain glucose metabolism, the effect might be small. Thus, we defined significance as instances when the minimum cluster size was 100 or more contiguous voxels, the uncorrected *p*-value was less than 0.001 at the voxel level, and the FWE corrected *p*-value was less than 0.05 at the cluster level. Coordinates of local maxima were converted from the MNI atlas to the Talairach space using the Talairach Client v2.4.3. A region-of-interest (ROI)-based analysis was conducted to validate the association between brain glucose metabolism, BMI, and osteocalcin. The mean uptake from the ROIs of each PET scan was extracted using an automated anatomical labeling template. The mean uptake was scaled to the global mean value for each PET scan. The scaled mean uptake was defined as the standardized uptake value ratio (SUVR).

### 2.4. Statistical Analysis

Multiple regression analysis was performed to validate which variables, including age, BMI, BMD T-score, and osteocalcin, showed an association with brain glucose metabolism presented as SUVR. Results were considered statistically significant when the *p*-value was less than 0.05. Data were analyzed using MedCalc^®^ Statistical Software version 20.111 (MedCalc Software Ltd., Ostend, Belgium; https://www.medcalc.org; 2022).

## 3. Results

### 3.1. Participants’ Characteristics

A total of 179 men were included in this study. The mean age, height, weight, BMI, fasting glucose level, HbA1C level, BMD, and osteocalcin level are summarized in Table 1. Among them, 64 participants were obese, with a mean BMI of 27.23 ± 1.77 kg/m^2^, and the remaining 115 participants were non-obese, with a mean BMI of 22.93 ± 1.50 kg/m^2^. The BMIs of the obese and nonobese participants were significantly different (*p* < 0.0001). BMI was negatively correlated with osteocalcin levels (r = −0.226; *p* = 0.0024). Three participants had DM. Seven participants showed either a high blood glucose level of ≥126 mg/dL or a high HbA1C level of ≥6.5%. Two patients had osteoporosis. Forty-three patients developed osteopenia. One hundred thirty-four participants had normal BMD.

### 3.2. Brain Region Showing Correlation with BMI and Osteocalcin

**Figure 1:**
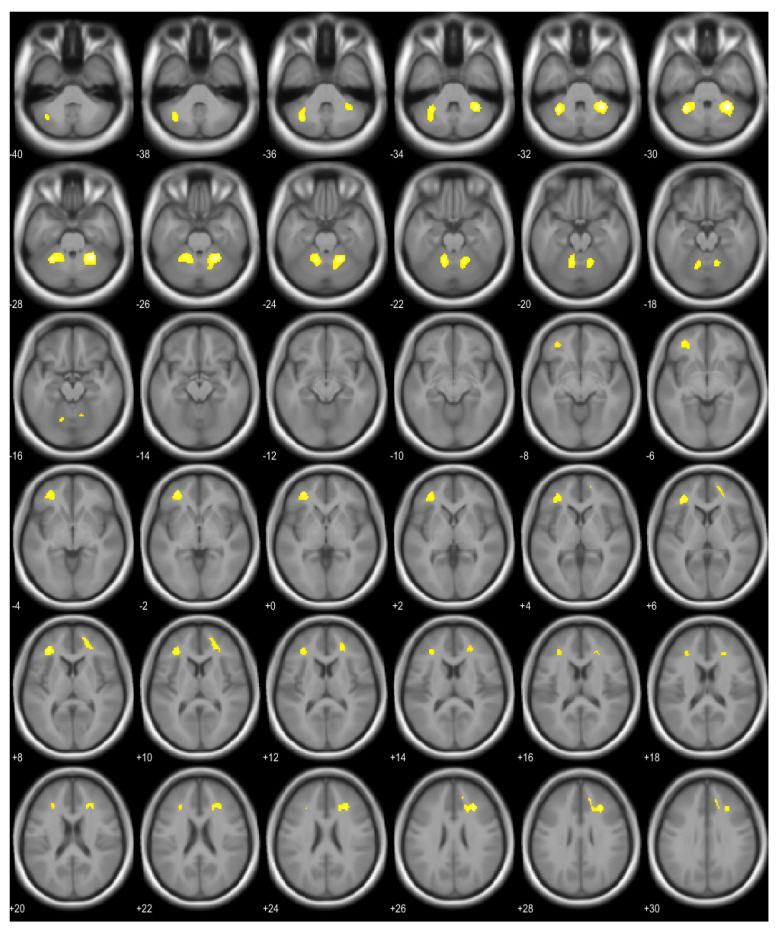
Brain regions showing positive correlations with body mass index (marked in yellow).

BMI was positively correlated with brain glucose metabolism in the anterior lobe of the right cerebellum, the anterior and posterior lobes of the left cerebellum, the right middle frontal gyrus (Brodmann area 9), the right cingulate gyrus (Brodmann area 32), the right anterior cingulate (Brodmann area 32), the left middle frontal gyrus (Brodmann area 10), and the subgyral area of the left frontal lobe (Figure 1 and Table 1).

**Figure 2:**
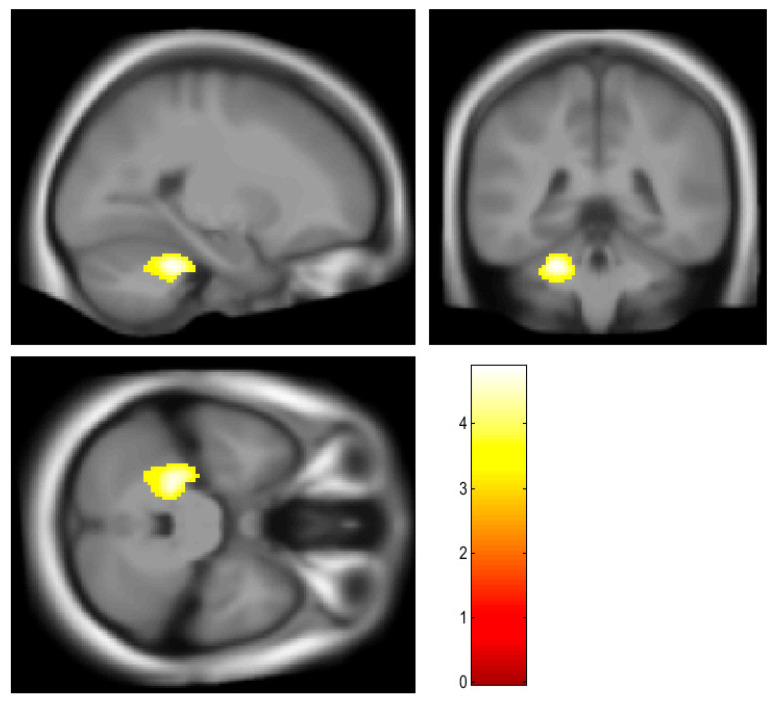
Brain regions showing negative correlations with osteocalcin (marked in yellow). Anterior lobe of the left cerebellum showed negative correlation with osteocalcin.

Osteocalcin was negatively correlated with glucose metabolism in the anterior lobe of the left cerebellum (Figure 2 and Table 2).

As BMD is known to correlate with osteocalcin levels [14], we also performed a regression analysis using BMD as a covariate in addition to age. When BMD T-score was included as a covariate, the results did not show a significant difference. In the ROI analysis, areas 3 and 4,5 of the left cerebellum were significantly negatively correlated with osteocalcin (*p* = 0.0384 and *p* = 0.0299, respectively) (Table 3 and Table 4).

## 4. Discussion

In this study, we have reported that BMI is positively correlated with brain glucose metabolism in the anterior lobe of the right cerebellum, the anterior and posterior lobes of the left cerebellum, the right middle frontal gyrus, the right cingulate gyrus, the right anterior cingulate, the left middle frontal gyrus, and the subgyral area of the left frontal lobe. Osteocalcin was negatively correlated with glucose metabolism in the left anterior lobe of the cerebellum.

Brodmann areas 9, 10, and 32, which correlated with brain glucose metabolism in this study, are part of the prefrontal cortex [15]. The prefrontal cortex plays a role in attention, working memory, decision making, and obesity [15,16]. The prefrontal cortex of obese individuals is known to have a lower gray matter volume and decreased connectivity [17], and decreased cognitive function, including attention, memory, and decision making, has been identified in obese individuals [18]. Volkow et al. reported a negative correlation between BMI and glucose metabolism in the prefrontal cortex [10]. They suggested that this result might have been due to impaired executive function [10]. A previous study using MRI reported that decreased gray matter volume and connectivity in the prefrontal cortex are associated with obesity [17]. Additionally, as F-18 FDG uptake reflects neuronal activity [19], the explanation provided in Volkow et al.’s study seems to be a reasonable hypothesis. However, Pegueroles et al. reported that a higher BMI was associated with higher cerebral FDG uptake in the left inferior temporal lobe, right insula, anterior cingulate, medial frontal, and orbitofrontal regions in healthy older participants, constituting results similar to the findings of this study [8]. They explained that this positive association between BMI and brain glucose metabolism might be due to neuroinflammation and astrogliosis [8]. Indeed, obesity is known to be related to gliosis [20,21], and several studies have reported that higher astrocytic reactivity is related to higher F-18 FDG uptake [22,23]. These conflicting results may be explained by sex. Estrogen is known to have protective effects against obesity and decrease proinflammatory cytokine levels [24,25,26]. Pegueroles et al. enrolled older healthy participants (85 men and 83 women, with a mean age of 73.5 years); however, only healthy men were enrolled in this study. However, a study by Volkow et al. enrolled healthy young participants (12 men and 9 women, with a mean age of 34 years). Thus, the inclusion of young women might have induced the conflicting results. Further studies are needed to evaluate the association between obesity, brain glucose metabolism, and sex hormones.

Alongside the cerebellum’s known role in motor control [27], it also plays a role in cognition [28]. Cognitive function in the cerebellum is known to be distributed in the lateral aspect of the cerebellum [28]. The left posterior hemisphere of the cerebellum is associated with visuospatial functions, and the right posterior hemisphere is associated with language functions [28]. Both posterior hemispheres of the cerebellum are associated with executive functions [28]. Similarly, the prefrontal cortex and cerebellum are associated with decreased gray matter volume [17] and glial activation [21] among obese individuals. Thus, the positive correlation between BMI and brain glucose metabolism can also be explained by gliosis.

Osteocalcin, a 46-amino-acid protein produced by osteoblasts, is a marker of osteoblast activity and bone formation [29,30]. However, in addition to bone-related functions, osteocalcin also plays a role in fat mass regulation, energy metabolism, male fertility, and cognition [30]. The effects of osteocalcin on brain development and cognitive function have been demonstrated in mice and humans. Osteocalcin-deficient mice were more passive and showed increased anxiety and decreased memory than wild-type mice [12], and the injection of osteocalcin could rescue the impaired behavioral changes in osteocalcin-deficient mice [12]. A positive correlation between osteocalcin and cognitive performance has been reported among elderly and obese patients [12]. Osteocalcin levels are negatively correlated with chronic inflammation [30,31]. As mentioned above, it has been postulated that obesity induces brain inflammation, which results in increased glucose metabolism; thus, the negative correlation between osteocalcin and cerebellar glucose metabolism might imply a protective effect of osteocalcin through reducing inflammation. Further studies are required to validate the anti-inflammatory effects of osteocalcin in the brain.

This study had several limitations. First, MRI was not performed on the enrolled participants. Thus, PET image processing and the possibility of hidden brain abnormalities might have affected the results. Second, neurocognitive function tests were not performed on the enrolled participants. Therefore, hidden neurodegenerative diseases might still have been present or developing. However, the mean age of the enrolled participants was relatively low, and they did not have any clinically diagnosed psychiatric diseases or cognitive dysfunctions. Therefore, it is highly unlikely that they were affected by psychiatric or neurodegenerative diseases.

## 5. Conclusions

In conclusion, BMI was positively correlated with glucose metabolism in the prefrontal cortex and cerebellum. Osteocalcin levels were negatively correlated with brain glucose metabolism in the left cerebellum.

## Figures and Tables

**Table 1:** Subjects’ characteristics.

Variable	Value
Number of subjects	179
Age	46.20 ± 6.22
Height (cm)	171.36 ± 4.99
Weight (kg)	71.93 ± 8.62
BMI	24.49 ± 2.64
Fasting glucose level (mg/dL)	92.97 ± 13.02
HbA1C	5.60 ± 0.54
BMD	
Lumbar spine T-score	−0.04 ± 1.14
Femur neck T-score	−0.06 ± 0.97
Femur total T-score	0.45 ± 0.90
Osteocalcin (ng/mL)	16.36 ± 5.08

Data are mean ± SD values; BMI: body mass index; HbA1C: glycated hemoglobin; BMD: bone mineral density.

**Table 2:** Brain regions showing positive correlations with body mass index.

Brain Area	Voxel	T	*p* (FWE Corrected)	Coordinates (x, y, z)
Right cerebellum, anterior lobe	521	6.55	<0.001	28, −48, −30
Left cerebellum, anterior and posterior lobe	522	6.19	<0.001	−30, −50, −30
Right middle frontal gyrus, right cingulate gyrus, right anterior cingulate	297	5.78	<0.001	28, 30, 26
Left middle frontal gyrus, sub-gyral area of left frontal lobe	368	5.66	<0.001	−34, 38, 8

FWE: family-wise error.

**Table 3:** Brain region showing negative correlation with osteocalcin.

Brain Area	Voxel	T	*p* (FWE Corrected for Cluster Level)	Coordinates (x, y, z)
Left cerebellum, anterior lobe	458	4.87	0.009	−24, −40, −30

FWE: family-wise error.

**Table 4:** Brain regions showing correlations with osteocalcin in multiple regression analysis.

Brain Area	Coefficient	S.E	t	*p*
Left cerebellum, 3	−0.005101	0.004714	−2.086	0.0384
Left cerebellum, 4_5	−0.002666	0.001218	−2.189	0.0299

S.E: Standard error.

## Data Availability

The data presented in this study are available on request from the corresponding author. The data are not publicly available due to restrictions associated with participants’ privacy.
